# Quality of life and work functionality in severe asthma patients: the impact of biological therapies

**DOI:** 10.1186/s12995-024-00406-9

**Published:** 2024-03-20

**Authors:** Veruscka Leso, Claudio Candia, Daniela Pacella, Antonio Molino, Caterina Nocera, Mauro Maniscalco, Ivo Iavicoli

**Affiliations:** 1 Department of Public Health, Section of Occupational Medicine, University of Naples Federico II, Via S. Pansini 5, Naples, 80131 Italy; 2 Department of Clinical Medicine and Surgery, University of Naples “Federico II”, Naples, 80131 Italy; 3 Department of Public Health, University of Naples Federico II, Via S. Pansini 5, Naples, 80131 Italy; 4 Pulmonary Rehabilitation Unit of Telese Terme Institute, Istituti Clinici Scientifici Maugeri IRCCS, Telese Terme, 82037 Italy

**Keywords:** Severe asthma, Asthma management, Lung functionality, Biological therapy, Occupational health, Quality of life, Work productivity, Work ability, Health promotion

## Abstract

**Background:**

Severe asthma can cause poor health status, poor health-related quality of life (HRQoL) and an impaired functioning at work. However, to date, limited data are available on the impact of the biological therapies on such outcomes. Therefore, aim of the present study was to prospectively assess the clinical, quality of life and work functionality issues in severe asthma patients both at baseline and after 6 months of biological therapies and determine which individual, pathological and occupational factors can influence such parameters.

**Methods:**

Fifty-two patients were enrolled between December 2022 and June 2023. Patients’ personal, clinical, functional and occupational features were assessed. The Short Form Health Survey (SF-12), the Work Productivity and Activity Impairment (WPAI) questionnaire and the Work Ability Index (WAI) were employed to assess HRQoL, the employee’s productivity and perception of work ability, respectively.

**Results:**

Among the enrolled patients, 30 (57.70%) were employed. Biological therapy induced a significant improvement in clinical and functional parameters, e.g., FEV1% (72 ± 12 vs.87 ± 13%; 72 ± 14 vs. 86 ± 14%), FVC% (92 ± 11 vs. 101 ± 11%; 90 ± 13 vs. 98 ± 14%) and FEV_1_/FVC (62 ± 11 vs. 71 ± 8%; 64 ± 9 vs. 70 ± 8%) in workers and non-workers, respectively (*P* < 0.001). Comparably, the perception of life quality significantly improved, as physical and mental health scores, in the overall cohort, increased from 40.7 ± 10.3 and 48.5 ± 8.5 to 46.8 ± 8.6 and 51.6 ± 6.4, respectively (*P* < 0.001). The work ability perception significantly improved from a moderate to a good one (34 ± 6 vs. 40 ± 6, *P* = 0.001). A significant reduction in the absenteeism (19 ± 15 vs. 3 ± 11%; *P* < 0.001) and presenteeism rate (53 ± 24 vs. 29 ± 26%; *P* < 0.001), and an improvement in daily (40 ± 27.5% vs. 28.9 ± 24.7%, *P* < 0.001, in the overall population) and work activities (57 ± 25 vs. 29 ± 27%, *P* < 0.001) was determined. Gender, age, symptoms control and pulmonary functionality were correlated with the physical and mental health perception, daily activity impairment and work ability.

**Conclusions:**

Our study pointed out that biological therapies improved clinical, general life and occupational outcomes in patients with severe asthma. The correlation between clinical aspects and psychological and occupational issues suggest the relevance for a multidisciplinary management of the disease for an effective participation of patients in the world of work.

## Background

Asthma is a heterogenous disease characterized by chronic airway inflammation. It is defined by respiratory symptoms, such as wheeze, shortness of breath, chest tightness and cough, that vary over time and in intensity, together with variable expiratory airflow limitations [1]. Asthma affects more than 300 million people worldwide and is one of the most common respiratory diseases [1, 2]. Up to 10% of adults with asthma suffer from a severe form of the disease. Such patients report persistent symptoms or frequent exacerbations that require repetitive glucocorticoid bursts, maintenance oral glucocorticoid therapy, or both, despite adequate treatment with high-dose inhaled glucocorticoids, long-acting β2-agonists, and long-acting muscarinic antagonists [3]. Add-on treatment, which may include biological therapies, is needed to reduce the burden of the disease.

Impacts of severe asthma include exacerbations, poor health status, poor health-related quality of life (HRQoL) and impairment in functioning at work and in other roles [4, 5]. Concerning the employment rate, lower percentages of severe asthma affected patients retained an employment compared to those with a non-severe form of the disease [6–9]. A worst work ability perception was reported by asthmatic patients compared to healthy controls, particularly in those with severe asthma [7, 10–13], diagnosed in adulthood [14], with comorbidities [10, 15], and exposed to occupational risk factors (e.g., airborne pollutants, high physical workload) that can function as triggers of asthma exacerbations [16, 17]. Regarding work productivity, a reduction was demonstrated in poorly controlled asthma compared to well-controlled one, particularly in terms of absenteeism, presenteeism as well as work and activity impairments [8, 9, 18–21]. Optimizing asthma control was reported to significantly improve presenteeism and absenteeism rates [6, 22–24].

A common goal for affected subjects and health care is that patients are empowered to live a life free of disease symptoms, to reduce the number of hospital and emergency care visits, the loss of workdays, and the constraints placed on their daily lives. In this view, according to an holistic approach to health, it seems important to obtain a comprehensive understanding of the impact that severe asthma might have on the quality of life and work functionality, as two inter-related areas contributing to the human health and well-being, and the possible improvements achieved through treatments to define suitable strategies for a successful management of the disease. Therefore, the aim of the present study was to assess individual, functional, pathological, and occupational factors affecting physical and mental health status of affected patients as well as their employment rate, productivity, and perception of work ability at baseline and after 6 months of biological therapies. This may provide evidence for the effectiveness of such therapeutically approach to improve clinical, personal and professional outcomes.

## Methods

### Participants and study design

Between December 2022 and June 2023, patients were prospectively enrolled among outpatients and inpatients attending the Respiratory Unit of the “Federico II” University Hospital - Monaldi Hospital, in Naples, Campania Region, Italy. Patients ≥ 18 years were considered eligible if they had a confirmed diagnosis of severe asthma, as an uncontrolled disease despite adherence with maximal optimized therapy and treatment of contributory factors or that worsens when high dose treatment is decreased [3]; required an add-on biological treatment according to guidelines and the Italian Drug Regulation Authority (AIFA) indications for prescription that was initiated immediately after the enrollment visit; and were able to provide voluntary, written informed consent to participate in the research. Exclusion criteria were incomplete diagnostic procedures; diagnosis of mild or moderate asthma; non-request or contraindications for add-on biological therapies; scarce compliance or adverse event to the biological treatment; incapacity or lack of willingness to provide written consent. The study protocol was reviewed and approved by the Ethics Committee of the University Hospital “Federico II” (n. 278/20). Whenever applicable, this study was conducted in accordance with the Strengthening the Reporting of Observational Studies in Epidemiology (STROBE) reporting guidelines [25].

### Sociodemographic and clinical features

Demographic, clinical and occupational information were collected from the enrolled patients. These included personal data, such as age, gender, body mass index (BMI); marital status, educational level and voluptuary habits (e.g., smoking and alcohol use). In addition, age at diagnosis, respiratory symptoms at enrolment, self-reported exacerbations occurred during the 12 months before the investigation, medical therapy, presence of comorbid conditions were addressed. All study procedures were performed at enrollment and at 6-month follow-up.

The individual’s asthma control was measured by the Asthma Control Test (ACT). The ACT [26, 27] is a brief, patient-reported assessment of asthma symptoms and impact typically used in clinical practice to monitor the effectiveness of asthma management and to support treatment decisions. The ACT consists of five items: (1) activity limitation, (2) shortness of breath, (3) awaking because of asthma symptoms, (4) use of reliever medication and (5) global judgment of asthma control. All items refer to the last 4 weeks and are scaled from 1 to 5. The sum indicates asthma control with scores of 25 meaning perfectly controlled asthma, scores > 19 indicating well-controlled disease, scores between 15 and 19 reflecting partially controlled and scores < 15 poorly controlled asthma, respectively [28].

### Functional parameters

A simple spirometry was performed at each time-point and reported according to the most recent American Thoracic Society/European Respiratory Society (ATS/ERS) guidelines [29], using the Master Screen Body® Jaeger–Carefusion spirometer (22745 Savi Ranch Parkway, Yorba Linda, CA, USA). The spirometry maneuvers were repeated consecutively up to a maximum of eight forced breaths until three acceptable and reproducible curves were obtained for each patient. All patients stopped the bronchodilator before the test, at least 6 h for short-acting bronchodilators and 12 for long-acting bronchodilators. The results of the pulmonary function tests (forced vital capacity [FVC], forced expiratory volume in the first second [FEV_1_] and the ratio FEV_1_/FVC) were registered both in absolute value and as percentage of predicted value.

All patients also underwent a blood analysis at enrolment and after 6 months after starting the biological therapy. Both absolute (n/μL) and percentage blood eosinophil counts (BECs) were assessed. The tests were conducted at the laboratory of the Clinical Biochemistry Unit, Monaldi Hospital, Naples. Additionally, fractional exhaled nitric oxide (FeNO) was measured using the Vivatmo-PRO device (Bosch, Waiblingen, Germany), an electrochemical NO detector. Briefly, the patient had to breathe through a mouthpiece and against resistance, maintaining a constant flow of approximately 50 ml/s for approximately ten seconds. At the end of the procedure, the detector expressed the value of FeNO in parts per billion (ppb). The test was considered positive if the result was greater than or equal to 25 ppb, according to the ATS guidelines [30].

### Occupational features

The sampling population was divided into employed and unemployed. This latter group included students, homemakers, people seeking a job and inactive persons (i.e., pensioners and those not seeking a job). Moreover, fixed term or permanent positions, as well as full-time or part-time schedules were assessed. The International Standard Industrial Classification of All Economic Activities was employed to classify the productive field of employment [31]. Jobs were divided in ten occupational activity families according to the International Standard Classification of Occupations established by the International Labour Organization [32]. The occupational risks experienced during specific job tasks, the health surveillance programs adopted in the workplace and the suitability of patients for work were also explored.

To measure impairments in work and activity, the Work Productivity and Activity Impairment (WPAI) questionnaire was administered at each time-point [33]. The WPAI questionnaire measures work time missed and work and activity impairment due to a specified health problem during the last 7 days. It consists of six questions which evaluate the following information: employment status, hours missed due to asthma, hours missed due to other reasons, hours actually worked, and the degree to which asthma affected productivity while working or regular activities (from 0 [no effect] to 10 [maximum impairment]). The sum of work time missed [‘absenteeism’] and impaired work time [‘presenteeism’] yields the overall work productivity loss score [6]. Unemployed patients only answered questions relating to employment status and daily activities.

The employee’s perception of work ability was assessed through the self-administered Work Ability Index (WAI) at enrolment and after 6 months of biological therapy [34, 35]. It is a summary measure of seven items including: individuals’ current ability to work in comparison with their best years of life,their ability to work concerning their demand for work; the number of diagnosed diseases or limitations from which they suffer; their estimated impairments due to diseases/abilities or limitations; the number of absent days they have taken during the previous year; self-prognosis of work ability for the next 2 years. The WAI score ranges from 7 to 49 points. Points of 7–27, 28–36, 37–43, and 44–49 correspond to low, moderate, good and excellent work ability, respectively [36].

### Health-related quality of life

HRQoL was measured with the 12-Item Short Form Health Survey (SF-12) at enrolment and after 6 months of biological therapy [37]. The SF-12 consists of 12 items on eight scales (“physical functioning”, “role limitations due to physical problems”, “bodily pain”, “general health”, “vitality”, “social functioning”, “role limitations due to emotional problems”, and “perceived mental health”). Items are combined and transformed, resulting in the physical component summary score (PCS-12) and mental component summary (MCS-12), both ranging from 0 (worst) to 100 (best).

### Statistical analyses

Data are presented as frequency (percentages) for the categorical variables, while they are presented as mean ± standard deviation (SD) for continuous variables. Χ^2^ test and Fisher’s test, as appropriate, were used to test for the differences among the groups for categorical variables. Student’s t-test or Mann–Whitney U-test were used to test for the differences among the groups for normally and nonnormally distributed continuous variables, respectively. The differences between before and after biological therapy were calculated with the Student’s T test for paired samples or with the Wilcoxon test for continuous variables, or with the Mc Nemar test for categorical variables. Linear regression model was used to predict WAI score using the reported variables, while logistic regression model was used to investigate the predictors of employment status. For the linear models, results are reported as estimates of the beta coefficients, which represent the estimated mean variation in the outcome variable for a unit increase in the predictor. Correlations between the WAI scores and the variables of interest were assessed using Pearson’s or Spearman correlation as appropriate. Normality of the distributions was assessed with the Shapiro-Wilk test. The significance level for all analyses was set to α = 0.05. All analyses were performed using the statistical software R (version 4.3.0).

## Results

### Characteristics of the study participants

**Fig. 1:**
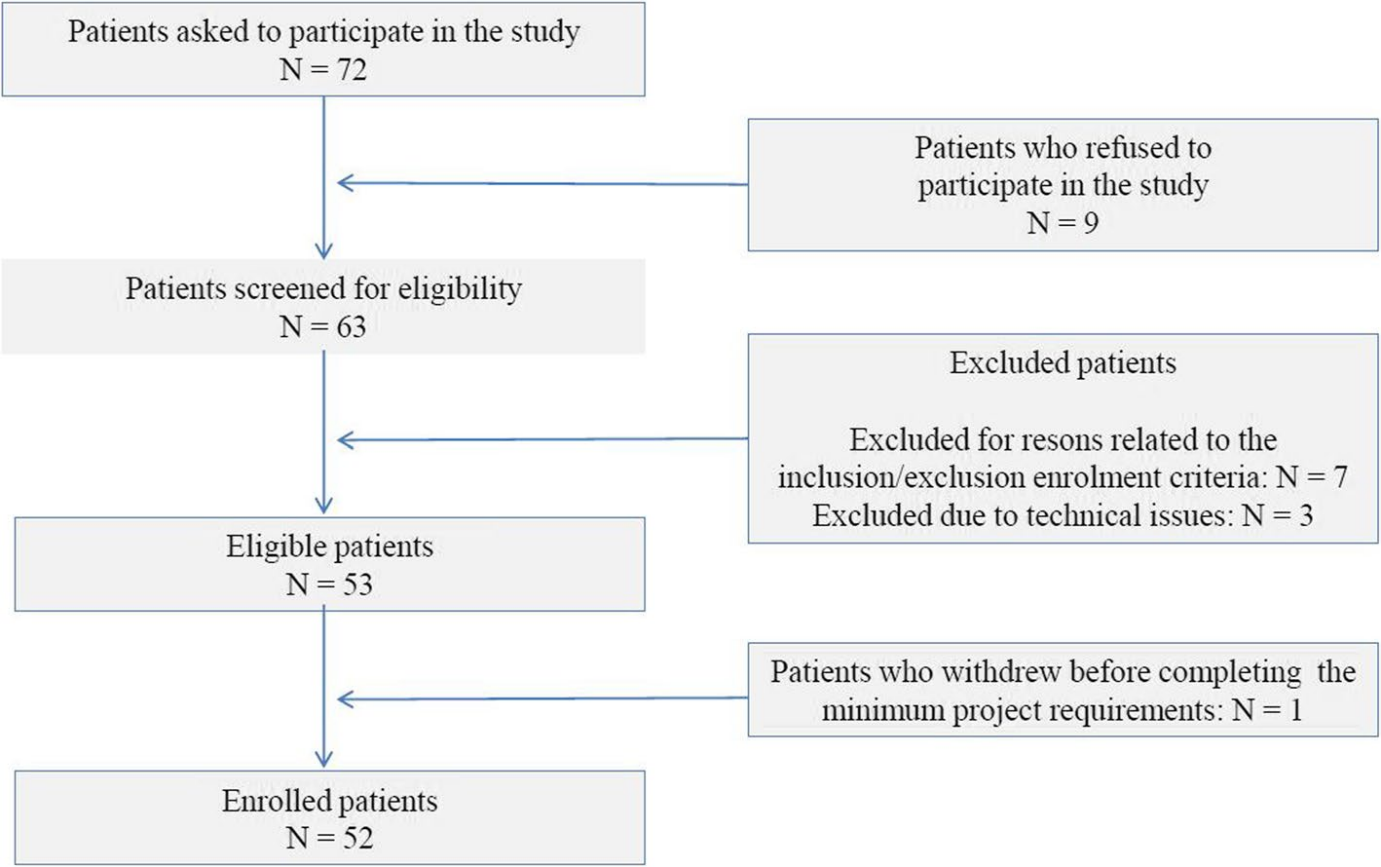
STROBE flow diagram of study participants’ enrolment

After excluding nine patients refusing to enter the study, 63 participants were screened for eligibility. Out of those, seven (11.1%) were excluded for reasons related to the inclusion/exclusion criteria of our protocol. Among the 56 eligible asthma patients, one (1.8%) withdrew before completion of the minimum project requirements, while three (5.4%) were excluded due to technical issues related to the study procedures. Therefore, a total of 52 patients entered the study (Fig. 1). The main baseline characteristics of the study participants have been listed in Table 1. Overall, a total of 26 (50%) patients were female, and the mean age of the study participants was 57.0 ± 11.7 years. The average BMI, 27.4 ± 3.4, highlighted a mean condition of overweight. Eighty-four percent of the population (*n* = 44) were non-smokers. In relation to the level of education, 21 (40%) and 23 (44%) patients had a primary or a secondary level of education, respectively. Concerning clinical features, the mean age at the diagnosis was 32.3 ± 12.6 years. All patients reported at least two acute exacerbations in the previous year, with an overall mean value of 2.90 ± 0.72. The mean ± SD eosinophil count detected by peripheral blood samples was 467.7 ± 213.1 per microliter of blood. The mean ACT score (11.6 ± 2.3) suggested a poor control of asthma symptoms. Spirometry parameters recorded at baseline, indicated a mild functional deficit, with mean ± SD of FEV_1_% of 71.7 ± 12.8% predicted and FVC% values of 91.1 ± 11.9% predicted. Mean FeNO levels (28.3 ± 5.3 ppb) were moderately elevated.

**Table 1:** Baseline characteristics of the overall study population by working and non-working groups

	**Study population *N* = 52**	**Non-workers, *N* = 22**	**Workers, *N* = 30**	***P*-value**
Age (M ± DS)	57 ± 11.7	61 ± 15	54 ± 9	0.057
Gender N (%)				**0.005**
F	26 (50%)	16 (73%)	10 (33%)	
M	26 (50%)	6 (27%)	20 (67%)	
BMI (M ± SD)	27.4 ± 3.4	28.4 ± 3.0	26.7 ± 3.7	0.071
Age at diagnosis (M ± SD)	32.3 ± 12.6	33 ± 13	32 ± 13	0.778
Smoking status N (%)				0.615
Non-smokers	44 (84%)	20 (91%)	24 (80%)	
Smokers	3 (6%)	1 (4.5%)	2 (6.7%)	
Ex-smokers	5 (10%)	1 (4.5%)	4 (13%)	
Education level N (%)				**0.028**
A	21 (40.4%)	10 (45%)	11 (37%)	
B	23 (44.2%)	12 (55%)	11 (37%)	
C	8 (15.4%)	0 (0%)	8 (27%)	
Exacerbations in the preceding 12 months (M ± SD)	2.90 ± 0.72	2.95 ± 0.79	2.87 ± 0.68	0.846
Atopy N (%)	15 (29%)	3 (14%)	12 (40%)	**0.038**
Nasal polyposis N (%)	22 (42%)	7 (32%)	15 (50%)	0.190
Drug N (%)				0.821
Benralizumab	17 (33%)	7 (32%)	10 (33%)	
Dupilumab	23 (44%)	9 (41%)	14 (47%)	
Mepolizumab	12 (23%)	6 (27%)	6 (20%)	
ACT (M ± SD)	11.6 ± 2.3	12.18 ± 1.92	11.20 ± 2.57	0.121
Spirometric values (M ± SD)				
FEV_1_ L	2.1 ± 0.6	1.93 ± 0.72	2.28 ± 0.52	0.057
FEV_1_%	71.7 ± 12.8	72 ± 14	72 ± 12	0.929
FVC L	3.4 ± 1.1	3.05 ± 1.15	3.72 ± 0.95	**0.031**
FVC %	91.1 ± 11.9	90 ± 13	92 ± 11	0.511
FEV1/FVC	63.1 ± 9.9	62 ± 11	64 ± 9	0.571
FeNO (M ± SD) (ppb)	28.3 ± 5.3	26.7 ± 2.5	29.4 ± 6.5	**0.044**
Eosinophils (M ± SD) (n/μL)	467.7 ± 213.1	483 ± 242	457 ± 193	0.677

Significant differences between workers and non-workers (*p* < 0.05) are shown in bold

*Abbreviations*: *A* Primary level of education (primary and lower secondary school), *B* Secondary level of education (upper secondary school or vocational diploma), *C* Tertiary level of education (bachelor’s degree and postgraduate degree), *ACT* Asthma Control Test, *BMI* Body mass index, *FEV1* Forced expiratory volume in the first second, *FVC* Forced vital capacity, *M* Mean, *SD* Standard deviation

### Occupational features

#### Overall sample

More than a half of the enrolled population declared to be employed at the enrolment (30 out of 52; 57.7%) and the same subjects reported to retain job after 6 months of biological therapy. None started to work following such period of treatment. Within the not-working group (*n* = 22), 11 participants (50%) were engaged in full-time domestic activities, 7 (32%) were retired, and 4 (18%) were unemployed and not seeking an employment at all. Most affected workers retained a full-time job (22; 73%), while 8 (27%) had a part-time employment. About half of the working population (*n* = 16) reported to have a permanent contract, eight (27%) had a fixed-term one, and six (20%) reported to work as freelance. The mean ± SD length of employment was 18 ± 12 years, with over 77% of the workers having been employed for over 15 years (*n* = 17), suggesting the capability of these affected employees to effectively retain jobs for long time. Most workers (67%) were engaged in the private sector. The main fields of employment were the trade (17%) and services (17%) (Table 2). In line with this result, the most represented working category was characterized by employees in commercial and service activities (*n* = 10; 33%), followed by intellectual, scientific, and highly specialized professions (*n* = 6; 20%). When workers were questioned about the occupational risks of their professional activity, the following: inappropriate postures (38.5%), manual handling of loads (21.1%), the use of video terminals (13.5%), and the shift work or night shifts (13.5%) were most frequently highlighted. Other occupational risk factors, including those potentially related to asthma manifestations, e.g., dust and chemical exposure, were reported in a more limited percentage of cases (2%). More than a half of the employed population (*n* = 18; 60%) was subjected to routine health surveillance programs. In terms of fitness for work, 78% (*n* = 14) of the monitored workers were deemed suitable for their job tasks, while the remaining received specific prescriptions to adhere while performing their work or faced limitations in their job tasks.

**Table 2:** Productive field of employment and occupational activities performed by employed severe asthma affected subjects

**Industrial classification (ISIC)**	**n (%)**
A. Agriculture; forestry and fishing	-
B. Mining and quarrying	-
C. Manufacturing	2 (7)
D. Electricity; gas, steam and air conditioning supply	-
E. Water supply; sewerage, waste management and remediation activities	-
F. Construction	3 (10)
G. Wholesale and retail trade; repair of motor vehicles and motorcycles	5 (17)
H. Transportation and storage	1 (3)
I. Accommodation and food service activities	3 (10)
J. Information and communication	-
K. Financial and insurance activities	3 (10)
L. Real estate activities	-
M. Professional, scientific and technical activities	-
N. Administrative and support service activities	-
O. Public administration and defence; compulsory social security	1 (3)
P. Education	4 (13)
Q. Human health and social work activities	3 (10)
R. Arts, entertainment and recreation	-
S. Other service activities	5 (17)
T. Activities of households as employers; undifferentiated goods- and services-producing activities of households for own use	-
U. Activities of extraterritorial organizations and bodies	-
**Occupation classification (ISCO-08)**	**n (%)**
1 – Managers	1 (3)
2 – Professionals	6 (20)
3 - Technicians and associate professionals	1 (3)
4 - Clerical support workers	2 (7)
5 - Service and sales workers	10 (33)
6 - Skilled agricultural, forestry and fishery workers	-
7 - Craft and related trades workers	3 (10)
8 - Plant and machine operators, and assemblers	2 (7)
9 - Elementary occupations	4(13)
10 - Armed forces occupations	1(3)

#### Working and non-working groups

The characteristics of the working and non-working groups are summarized in Table 1. Workers were generally male (*P* = 0.005) and younger compared to non-working ones (*P* = 0.057) with a significantly higher prevalence of atopic subjects (*P* = 0.038). Concerning the education level, a significantly different distribution was determined as a greater percentage of subjects with a degree or post-graduate degree was represented among employed individuals compared to the unemployed ones (*P* = 0.028). No significant variations were determined with respect to baseline clinical parameters, apart from a significantly higher mean absolute FVC level in employed patients compared to non-workers (3.72 ± 0.95 L *vs.* 3.05 ± 1.15 L; *P* = 0.031), although values were in the normal range in both groups. FeNO values were significantly higher in the working group compared to the non-working one (29 ± 7 ppb *vs.* 26.7 ± 2.5 ppb; *P* = 0.04).

### Effects of the biological therapy on clinical, laboratory and functional variables

**Fig. 2:**
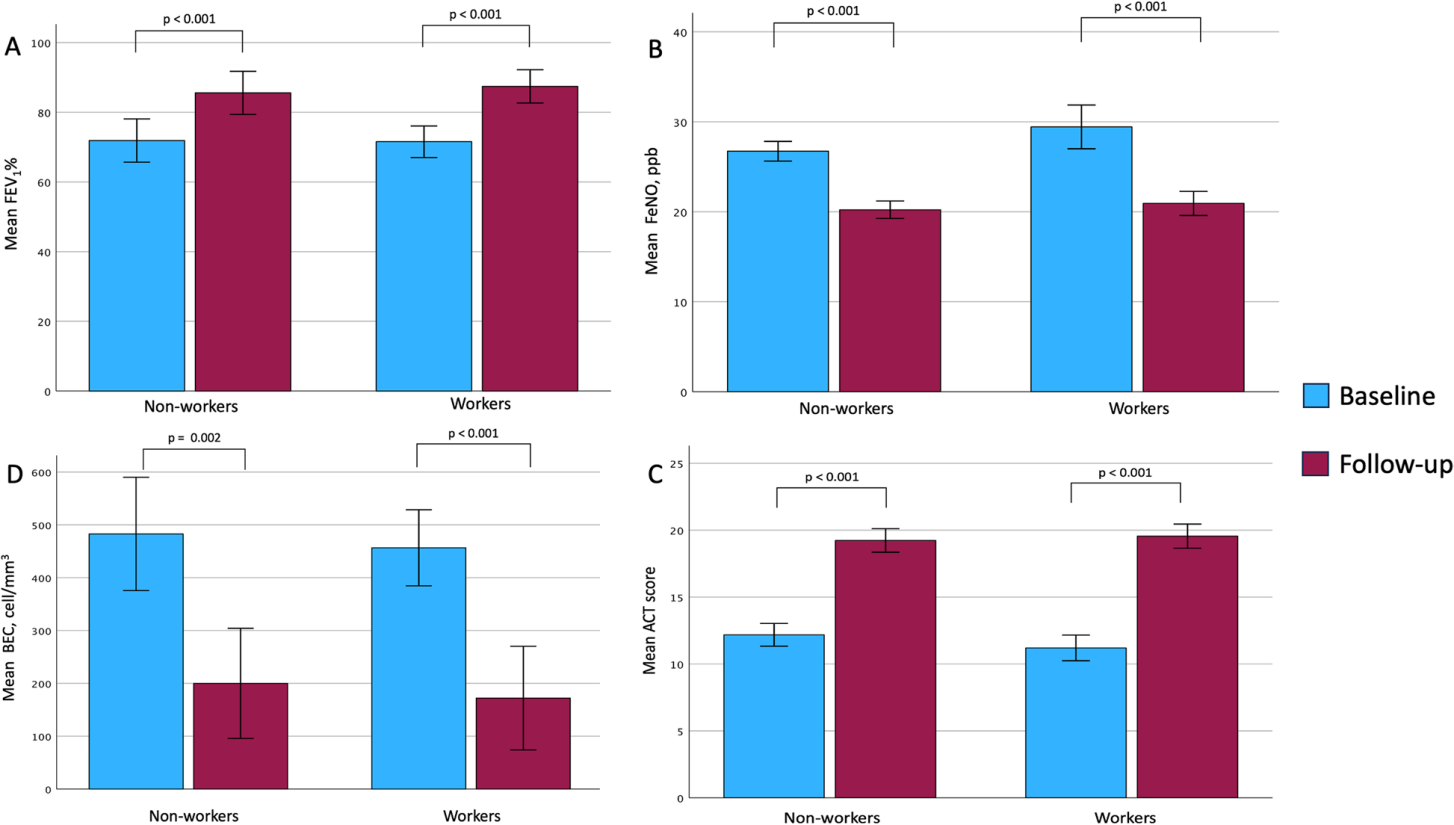
Mean clinical and functional outcomes before and after 6 months of biologic therapy. Mean clinical and functional outcomes before and after treatment (data reported as mean and 95% confidence interval). **A** Variation in FEV1%. **B** Variation in FeNO. **C** Variations in ACT; **D** Variation in BEC. Abbreviations: FEV_1_%, forced expiratory volume in the first second, percentage of predicted values; ACT, asthma control test; BEC, blood eosinophil count; FeNO: fractional exhaled nitric oxide

All patients included in the study received a full 6-month treatment and were compliant to the therapy. The individual total number of administrations were as follows: four for those who were prescribed benralizumab, six for those who were prescribed mepolizumab, and 13 for those who were prescribed dupilumab. A significant improvement in lung function parameters, i.e., in absolute FEV_1_ and FVC levels, as well as in FEV_1_%, FVC% was detected at the 6-month follow up in both groups (Table 3, Fig. 2). Comparably, the FEV_1_/FVC ratio changed from baseline levels indicating an airway flow obstruction, to mean values reaching proximity to the range of normal values after 6 months (*p* < 0.001). In both groups it was possible to observe a significant reduction in the biomarkers of T2-high inflammation, namely FeNO and BEC, expressed both as absolute and relative values. Finally, variations in the ACT score demonstrated a significant improvement in symptom control in both groups, reaching values within the ‘control’ range in both groups after 6 months of treatment. No significant difference was found when stratifying the patients according to the prescribed biological drug in use, possibly due to the small size of each subgroup.

**Table 3:** Differences before and after 6- months of biological drug therapy

**Features**	**Workers**			**NON-Workers**		
	**Enrolment, *N* = 30**	**Six months after enrolment, *N* = 30**	***p*-value**	**Enrolment, *N* = 22**	**Six months after enrolment, *N* = 22**	***p*-value**
Exacerbations	2.87 ± 0.68	0.27 ± 0.45	**< 0.001**	2.95 ± 0.79	0.27 ± 0.55	**< 0.001**
ACT	11.2 ± 2.6	19.6 ± 2.3	**< 0.001**	12.2 ± 1.9	19.2 ± 2.0	**< 0.001**
Spirometric values (M ± SD)						
FEV_1_ (L)	2.28 ± 0.52	2.80 ± 0.65	**< 0.001**	1.93 ± 0.72	2.30 ± 0.81	**< 0.001**
FEV_1_ (%)	72 ± 12	87 ± 13	**< 0.001**	72 ± 14	86 ± 14	**< 0.001**
FVC (L)	3.72 ± 0.95	4.03 ± 1.05	**0.002**	3.05 ± 1.15	3.30 ± 1.17	**0.001**
FVC%	92 ± 11	101 ± 11	**< 0.001**	90 ± 13	98 ± 14	**0.002**
FEV_1_/FVC	62 ± 11	71 ± 8	**< 0.001**	64 ± 9	70 ± 8	**< 0.001**
FeNO (ppb)	29 ± 7	21 ± 3	**< 0.001**	26.7 ± 2.5	20.2 ± 2.2	**< 0.001**
Eosinophils (n/μL)	457 ± 193	181 ± 258	**< 0.001**	483 ± 24	200 ± 235	**0.002**
Eosinophils %	5.7 ± 2.8	2.2 ± 3.2	**< 0.001**	5.96 ± 2.81	2.28 ± 2.67	**< 0.001**
PCS 12	36 ± 9	47 ± 9	**< 0.001**	32 ± 8	46 ± 8	**< 0.001**
MCS 12	47 ± 8	52 ± 7	**< 0.001**	44 ± 11	51 ± 6	**0.003**
WAI	34 ± 6	40 ± 6	**< 0.001**			
Absenteeism (%)	19 ± 15	3 ± 11	**< 0.001**			
Presenteeism (%)	53 ± 24	29 ± 26	**< 0.001**			
Overall work impairment (%)	57 ± 25	29 ± 27	**< 0.001**			
Impact on daily activities (%)	41 ± 23	21 ± 21	**< 0.001**	65 ± 21	40 ± 26	**< 0.001**

Significant differences (*p* < 0.05) are shown in bold

*Abbreviations*: *ACT* Asthma Control Test, *FEV*_*1*_ Forced expiratory volume in the first second, *FVC* Forced vital capacity, *PCS* Physical component summary, *MCS* Mental component summary, *WAI* Work ability index

### Quality of life

Biological therapy significantly improved the perception of life quality in the overall population, as well as in working and non-working groups. In fact, in the total number of enrolled subjects, mean SF12 scores ± SD increased from 40.7 ± 10.3 and 48.5 ± 8.5 for physical and mental health to 46.8 ± 8.6 and 51.6 ± 6.4, respectively (*P* < 0.001). After biological treatments, in the working group, scores for PCS and MCS improved from 33 ± 8 to 47 ± 9 (*P* < 0.001) and from 44 ± 11 to 52 ± 7 (*P* < 0.001), respectively (Table 3). Similarly, PCS values increased from 36 ± 9 to 46 ± 8 (*P* < 0.001) and MCS from 47 ± 8 to 51 ± 6 (*P* = 0.003) in unemployed subjects. No significant differences emerged between the two groups at both the time points.

### Effects of biological therapy on work ability and productivity impairment

**Fig. 3:**
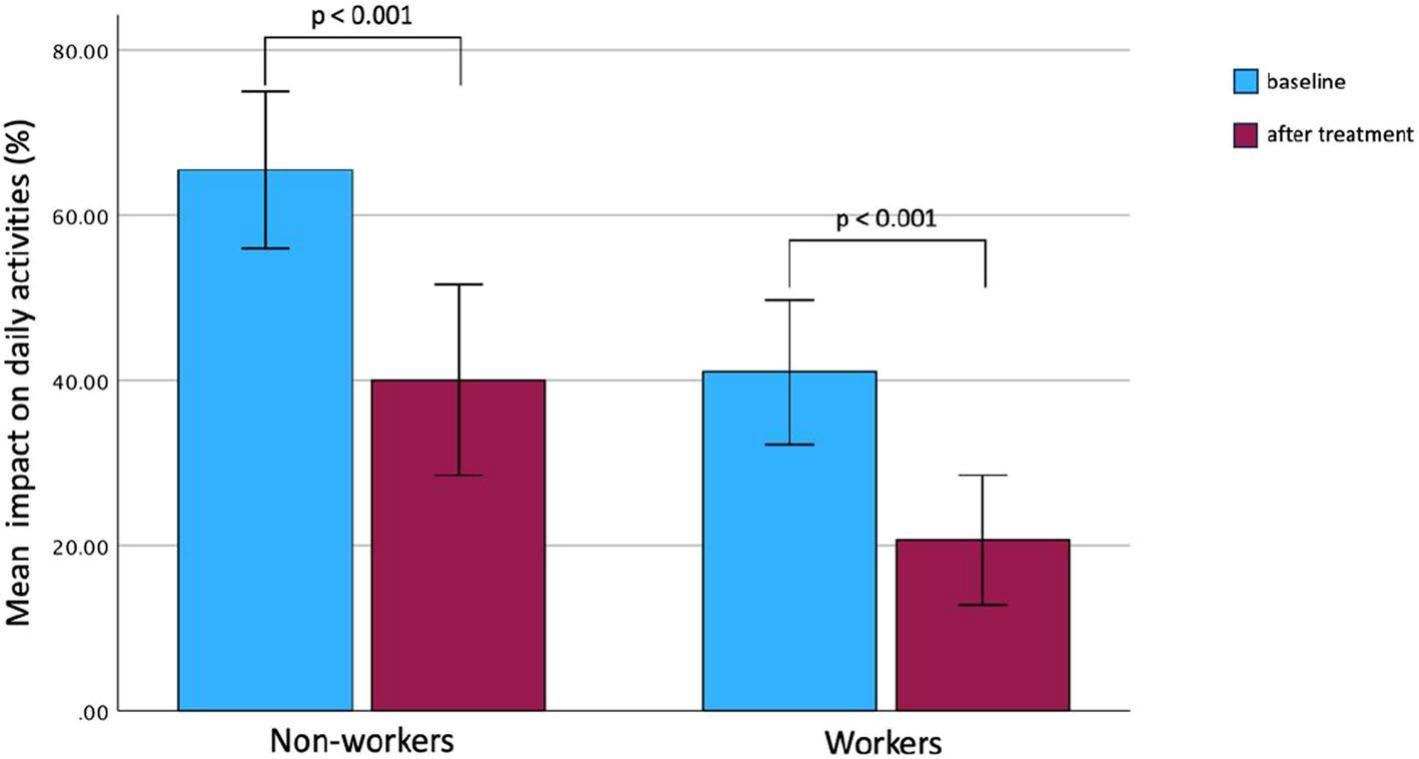
Impairment of daily activities in both workers and non-workers before and after 6 months of biologic therapy. Data reported as mean and 95% confidence interval

Six months of biological treatment significantly increased the average perception of work ability (*n* = 30) from a moderate level to a good one (mean ± SD WAI score: 34 ± 6 vs. 40 ± 6, *P* = 0.001) (Table 3). In the entire cohort, the disease caused impairment in carrying out daily activities, although a significant improvement was evident after 6 months of biological therapies (40 ± 27.5% vs. 28.9 ± 24.7; *P* < 0.001). Such a significant improvement was evident also when the working (41 ± 23% vs. 21 ± 21%; *P* < 0.001) and non-working subgroup (65 ± 21% vs. 40 ± 26%; *P* < 0.001) were separately analysed. Interestingly, a significantly (*P* < 0.001) greater daily activity impairment was evident in unemployed subjects both at the baseline and at 6 months after the add-on treatment (Table 3; Fig. 3).

**Fig. 4:**
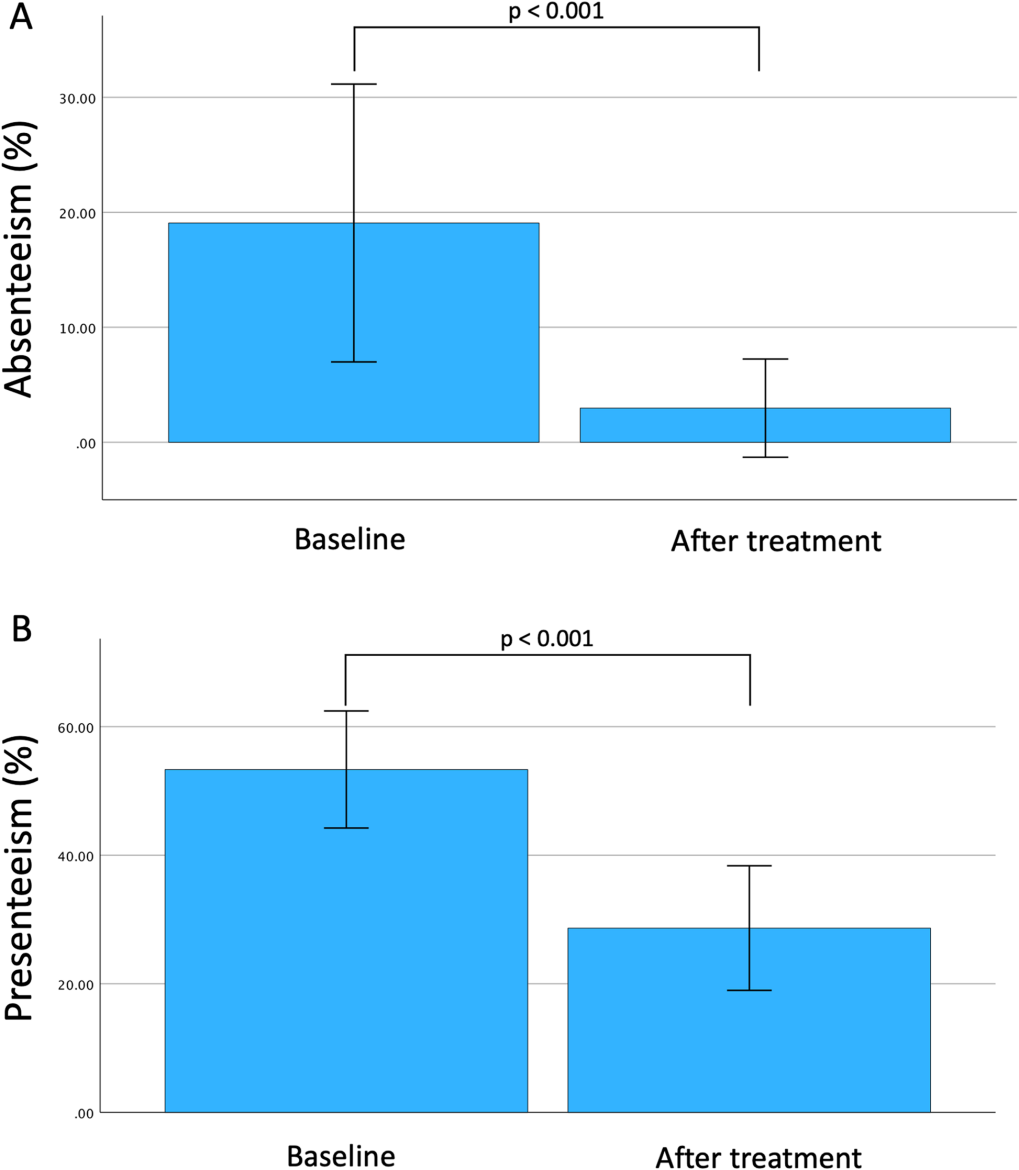
Absenteeism (**A**) and presenteeism (**B**) rate before and after 6 months of biologic therapy. Data reported as mean and 95% confidence interval

A mean 19 ± 15% percentage of absenteeism was reported at the enrolment, with a significant reduction after 6 months of treatment, reaching an average percentage of 3 ± 11% (*P* < 0.001) (Table 4). As regards presenteeism, the results obtained showed a mean ± SD percentage equal to 53 ± 24% at the enrolment and 29 ± 26% 6 months afterwards (*P* < 0.001) (Fig. 4), consistent with a significant improvement in all workers’ clinical and functional variables. Such a positive impact of the biological therapy could be demonstrated also by the significant reduction in work impairment observed at the two time points considered (57 ± 25% vs. 29 ± 27%; *P* < 0.001) (Table 3).

**Table 4:** Factors associated with the quality-of-life perception and daily activity impairment at the enrolment

**Features**	**N**	**Physical health perception (PCS)**			**Mental Health perception (MCS)**			**Daily Activity impairment**		
		**Beta**	**95% CI**	***p*-value**	**Beta**	**95% CI**	***p*-value**	**Beta**	**95% CI**^***1***^	***p*-value**
Gender	52									
F		-	-		-	-		-	-	
M		5.3	0.90, 9.8	**0.019**	1.4	-3.8, 6.5	0.594	-18	-31, -4.7	**0.009**
Age	52	-0.18	-0.37, 0.01	0.064	-0.20	-0.41, 0.01	0.065	0.17	-0.44, 0.77	0.581
Age at diagnosis	52	0.03	-0.15, 0.21	0.743	0.04	-0.16, 0.24	0.688	-0.24	-0.80, 0.32	0.388
BMI	52	0.37	-0.30, 1.0	0.275	-0.61	-1.3, 0.13	0.102	0.86	-1.2, 2.9	0.411
Smoke	52	-2.8	-6.5, 0.92	0.136	1.3	-2.9, 5.4	0.546	0.13	-11, 12	0.983
Education level	52									
A		-	-		-	-		-	-	
B		1.2	-3.5, 5.8	0.615	3.3	-2.0, 8.6	0.216	2.5	-13, 18	0.747
C		10	4.1, 17	**0.002**	9.5	2.2, 17	**0.012**	-4.7	-26, 17	0.663
Atopy	52	0.91	-4.3, 6.1	0.724	1.4	-4.3, 7.1	0.619	-1.9	-18, 14	0.811
Nasal polyposis	52	3.6	-1.0, 8.2	0.124	0.93	-4.3, 6.2	0.722	-7.8	-22, 6.5	0.276
Drug	52									
Benralizumab		-	-		-	-		-	-	
Dupilumab		4.4	-0.85, 9.6	0.099	3.2	-2.7, 9.1	0.282	-8.3	-25, 8.1	0.314
Mepolizumab		-1.0	-7.2, 5.1	0.734	0.11	-6.9, 7.1	0.974	-1.1	-21, 18	0.908
Exacerbations in the previous 12 months	52	-1.5	-4.8, 1.7	0.350	-2.3	-5.9, 1.2	0.193	14	4.6, 23	**0.004**
ACT	52	-0.08	-1.1, 0.92	0.867	1.5	0.43, 2.5	**0.006**	-2.9	-5.9, 0.07	0.055
Spirometric values (M ± SD)										
FEV_1_ (L)	52	6.4	3.2, 9.7	**< 0.001**	5.7	1.9, 9.5	**0.004**	-16	-26, -5.3	**0.004**
FEV_1_ (%)	52	0.16	-0.02, 0.34	0.079	0.33	0.15, 0.51	**< 0.001**	-0.51	-1.1, 0.03	0.064
FVC (L)	52	3.6	1.6, 5.5	**< 0.001**	1.7	-0.71, 4.0	0.165	-9.6	-16, -3.5	**0.003**
FVC%	52	0.14	-0.06, 0.33	0.169	0.28	0.08, 0.49	**0.007**	-0.82	-1.4, -0.26	**0.005**
FEV_1_/FVC	52	-0.01	-0.25, 0.23	0.932	0.31	0.07, 0.56	**0.013**	0.11	-0.62, 0.83	0.768
FeNO	52	0.17	-0.27, 0.61	0.435	0.00	-0.49, 0.48	0.988	0.01	-1.3, 1.4	0.992
Eosinophils (n/μL)	52	0.00	-0.01, 0.01	0.933	0.00	-0.01, 0.01	0.806	-0.01	-0.04, 0.03	0.763
Eosinophils %	52	0.14	-0.71, 1.0	0.737	-0.25	-1.2, 0.69	0.600	-0.23	-2.8, 2.4	0.862
Employment	52									
No		-	-		-	-		-	-	
Yes		3.4	-1.2, 8.1	0.144	3.1	-2.1, 8.3	0.233	-24	-37, -12	**< 0.001**

Significant associations (*p* < 0.05) are shown in bold

*Abbreviations*: *PCS* Summary of physical components, *MCS* Summary of mental components, *A* Primary education level (primary and lower secondary school), *B* Secondary education level (upper secondary school or vocational diploma), *C* Tertiary education level (bachelor’s degree and postgraduate degree), *ACT* Asthma Control Test, *BMI* Body mass index, *FEV1* Forced expiratory volume in the first second, *FVC* Forced vital capacity

### Factors associated with quality of life, daily activities and work functionality

A linear regression analysis was employed to identify possible factors influencing personal and occupational outcomes in affected patients. The self-perception of physical health was positively associated with the male gender, implying a tendency for men to report a more favourable assessment of their physical well-being (β = 5.3; 95%CI: 0.90, 9.8; *P* = 0.019) (Table 4). Comparably, a positive association was detected for a more advanced level of education (undergraduate, graduate or post-graduate) and a better perception of both physical (β = 10; 95%CI: 4.1, 17; *P* = 0.002) and mental health status (β = 9.5; 95%CI: 2.2, 17; *P* = 0.012). Concerning clinical parameters, a better symptom control was identified as a factor positively associated with the mental state of affected subjects. Additionally, the pulmonary function parameters, specifically FEV_1_ (β = 6.4; 95%CI: 3.2, 9.7; *P* < 0.001) and FVC (β = 3.6; 95%CI: 1.6, 5.5; *P* < 0.001), demonstrated a positive association with physical health status, suggesting their role as significant predictors for this parameter. Simultaneously, mental health status showed a positive association with FEV_1_, FEV_1_%, and FVC%.

Regarding the impairment in daily activities (Table 4), in line with the above-mentioned results, an inverse correlation was determined for the male gender (β = -18; 95%CI: -31, -4.7; *P* = 0.009), while the frequency of exacerbations in the 12 months prior to enrolment was positively associated (β = 14; 95%CI 4.6, 23; *P* = 0.004). Pulmonary function parameters, specifically FEV_1_ (β = -16; 95%CI: -26, -5.3; *P* = 0.004), FVC (β = -9.6; 95%CI: -16, -3.5; *P* = 0.003) and FVC% (β = -0.82; 95%CI: -1.4, -0.26; *P* = 0.005), were inversely associated to this impairment, suggesting that better respiratory function may be related to less difficulties in daily activities. Regarding the occupational status, to retain an employment was inversely associated to the daily activity impairment (β = -24; 95%CI: -37, -12; *P* < 0.001), in line with the above reported percentages of impairment found in the employed and non-employed groups.

Work ability was inversely associated with age (β = -0.29; 95%CI: -0.55, -0.03; *P* = 0.033), suggesting that increasing age was associated with a decline in the WA perception (Table 5). Conversely, the level of education was positively associated with WA (β = 7.4; 95%CI: 1.9–13; *P* = 0.010), supporting how a better level of education could be related to the possibility of being successfully employed in professional activities. In relation to the pulmonary function parameters, FEV_1_% was the only factor positively associated to WA (β = 0.22; 95%CI: 0.04, 0.40; *P* = 0.020). No personal characteristics or pathology-related factors emerged as significantly associated with the investigated occupational outcomes: absenteeism, presenteeism, and work activity impairment.

**Table 5:** Predictive factors of work ability perception at the enrolment

**Features**	**N**	**Beta**	**95% CI**	***P*-value**
Gender	30			
F		-	-	
M		0.95	-4.1, 6.0	0.705
Age	30	-0.29	-0.55, -0.03	**0.033**
Age at diagnosis	30	-0.02	-0.20, 0.17	0.845
BMI	30	0.22	-0.44, 0.88	0.504
Smoke	30	0.05	-3.4, 3.5	0.979
Education level	30			
A		-	-	
B		1.7	-3.3, 6.8	0.487
C		7.4	1.9, 13	**0.010**
Atopy	30	0.14	-4.8, 5.0	0.954
Nasal polyposis	30	-2.0	-6.7, 2.7	0.396
Drug	30			
Benralizumab		-	-	
Dupilumab		1.3	-4.2, 6.8	0.635
Mepolizumab		-1.3	-8.2, 5.5	0.693
Relapses in the previous 12 months	30	-0.57	-4.2, 3.0	0.747
ACT	30	0.31	-0.63, 1.3	0.502
Spirometric values (M ± SD)				
FEV_1_ (L)	30	3.8	-0.69, 8.2	0.094
FEV_1_ (%)	30	0.22	0.04, 0.40	**0.020**
FVC (L)	30	1.1	-1.4, 3.7	0.362
FVC%	30	0.16	-0.05, 0.36	0.132
FEV_1_/FVC	30	0.10	-0.13, 0.32	0.386
FeNO (ppb)	30	0.03	-0.35, 0.40	0.879
Eosinophils (n/μL)	30	0.01	0.00, 0.02	0.144
Eosinophils %	30	0.55	-0.30, 1.4	0.193
Current work duration (years)	30	-0.04	-0.24, 0.16	0.677
Type of contract	30			
Permanent		-	-	
Fixed-term		-3.4	-9.0, 2.1	0.212
Freelance		2.2	-3.9, 8.3	0.468
Sector	30			
Private		-	-	
Public		-1.1	-6.2, 4.0	0.661

Significant associations (*p* < 0.05) are shown in bold

*Abbreviations*: *A* Primary level of education (primary and lower secondary school), *B* Secondary level of education (upper secondary school or vocational diploma), *C* Tertiary level of education (bachelor’s degree and postgraduate degree), *BMI* Body mass index, *ACT* Asthma Control Test, *FEV*_*1*_ Forced expiratory volume in the first second, *FVC* Forced vital capacity

## Discussion

This research aimed to comprehensively assess the impact of severe asthma on the quality of life and work functionality of affected patients, before and after 6 months of treatment with biological therapies. It also had the purpose to identify individual, pathological, and occupational factors that may influence patients’ perception of physical and mental health status, as well as their active and effective participation in the labour force.

Concerning the employment status, more than a half of our investigated population retained a full- or part-time job at the time of the survey. This rate is slightly lower compared to the 63.2% reported in the second quarter of 2023 for the general Italian population of comparable age (50–64 years), but higher than the 51.6% reported for the South of Italy [38]. This suggests that severe asthma does not prevent patients’ employability. Unfortunately, in our study, no comparison could be done with an equivalent group of healthy matched individuals. With respect to the international scenario, our employment rate was in line with the percentages of 46%-61% reported in previous studies on severe asthma [6–8]. However, an appropriate comparison of the results can be biased by differences in social, economic and enrolment organization between countries, and also by the diverse years in which the investigations were performed.

In the employed subgroup, a significantly higher percentage of males was present (67%), well reflecting the different gender-related employment rate registered for males (67.4%) and female subjects (36.7%) in the South of Italy for the second quarter of 2023 [38]. This may be due to the generally greater involvement of women in household chores. Moreover, in line with literature data [39], unemployed subjects were generally, although not significantly, older than employed ones, maybe due to the greater opportunity to find work in a younger age. In addition, the finding of a lower age at diagnosis in the working subjects, although not significant, could be related to the fact that an early asthma diagnosis would lead affected individuals to a better adaptation to the pathology and more appropriate management, favoring their participation in the labor force and maintenance of appropriate employment. Additionally, it cannot be ruled out that a better socio-economic condition, due to the employment status, could be related to a greater likelihood to seek care and get diagnosed earlier. A higher level of education could be a key factor for a successful professional insertion as this could allow patients to achieve tertiary, less physically demanding positions, more easily adaptable to their health conditions in terms of work environments and organizational autonomy [9, 11, 12, 39].

Concerning clinical parameters, mixed results have been obtained. The employed subjects had significantly higher levels of FVC expressed in liters, suggesting that a better respiratory functionality may be a predictive factor for an effective workforce participation. However, this result should be interpreted with caution, as the FVC% predicted was not significantly different between the two groups supporting the possibility that the above-mentioned differences could be primarily related to the diverse age of the groups and require additional studies to be confirmed. Moreover, workers had a higher concentration of the FeNO inflammatory marker in the exhaled air. Also in this case, several variables, including inflammatory patterns underlying asthma [40], viral respiratory infections responsible for asthma flare-ups [41], and the smoking habit [42] can affect the results of this parameter and need to be verified in future research. With respect to the quality of life, no significant differences emerged between working and non-working patients, both referring to the physical and mental health status. In line with the idea that unemployment or not being able to work could be a negative predictor for the performance of common daily activities [43], an impairment was most frequently determined in female subjects, more represented in the non-working group. Moreover, an inverse relationship was determined between the employment status and the impairment in daily activities, suggesting how occupational engagement could be related to a better management of extra-occupational activities through a generally more active life approach. This finding suggests how an interdisciplinary management of the pathology, including an effective participation in the workforce and promotion of work ability, may act as an additional positive factor on the mental state of affected patients, contributing to their broader well-being. A higher education attainment was positively correlated with a better quality of life, perception of work ability and inversely correlated with productivity impairment. Such a level of education, in fact, would allow the attainment of professional positions, primarily in the tertiary sector, that would provide economic security and personal satisfaction, and more compatible with the health conditions of the affected workers, thus supporting the overall well-being of affected individuals [8, 44].

Specifically focusing on the impact of the biological therapy, the significant increase in the quality-of-life assessment scores after biological treatment, in working and non-working groups, suggested that a better symptoms’ control could significantly improve the perception of individual physical and mental health status. In particular, the positive association between the symptoms’ control and the perception of mental health status, underscores the importance to achieve an adequate management of the disease to assure patients and contribute to their overall mental well-being [45].

With respect to overall impairment in work productivity due to severe asthma, several studies confirmed a higher rate of absenteeism in affected subjects compared with healthy controls [7–11, 14, 24, 45–47]. Our study showed an absenteeism rate of 12%, with a significant improvement after biological therapy (3%). No personal or clinical factors emerged as predictive of higher levels of absenteeism, although the limited number of enrolled subjects limits the statistical power of our analyses. Compared with absenteeism, presenteeism is a relatively new and often underestimated indicator of workplace impairment [48]. The impact on work activities due to severe asthma was recognized to be primarily driven by a reduced productivity during work rather than absenteeism [49]. A significant reduction in the presenteeism rate could be determined after 6 months of biological therapy (53% vs. 29%). This highlights the importance to assess presenteeism when considering the economic burden of asthma and the overall work impairment, as it represents an important preventable burden through the adoption of workplace preventive and protective measures.

Although this work has comprehensively investigated the impact of severe asthma on patients’ working lives using validated questionnaires, also in relation to the biological therapy performed, several limitations need to be considered, in order also to design future studies to confirm and implement the obtained results. First, the observational and single-center organization of the study, together with the absence of a control group not treated with biological therapies prevent definite conclusions on the real role of such treatment on the quality of life and occupational outcomes investigated. Future research should include a greater number of subjects with severe asthma, in longitudinal and longer follow-up assessment to ascertain the real benefits of the biological therapy undertaken with respect to traditional approaches. Additionally, a multi-centered research methodology should be pursued to obtain data that may be representative also of welfare, economic status, work organization and cultural aspects different between countries. Moreover, as the absenteeism, presenteeism and activity impairment evaluation, performed through the WPAI, referred to a period of 7 days before the survey, it could be useful to plan future investigations with closer scheduled times of assessment to better follow up such parameters of work productivity. Our study population presented with an elevated BEC at baseline, which might be the result of a selection bias. In fact, we enrolled mostly middle-aged adults with severe asthma, among whom the late-onset non-allergic eosinophilic endotype is quite common [50]. The current study design did not allow to assess why asthmatic individuals dropped out or failed to enter the workforce, particularly how much the unemployment status was related to the underlying disease. Knowing such reasons could enable interventions aimed at promoting and supporting patients in labor force participation. An additional aspect to be considered for a correct interpretation of the results is the possible impact that the SARS-CoV-2 pandemic may have exerted on the employment status of the general population, and in particular of those who suffered for health-related difficulties in being engaged and in maintaining a job activity. Future studies should be also aimed to evaluate the quality of life and occupational issues of affected patients in comparison to healthy controls to achieve a more accurate multifaced picture of the disease impact on the patients’ life. Finally, further studies are necessary to confirm factors predictive for changes in individuals’ perception of quality of life, work ability or productivity to define appropriate strategies for managing affected patients at work.

## Conclusions

Our study pointed out that patients with severe asthma require a multidisciplinary management that takes into account not only clinical aspects, but also psychological and occupational issues, including educational choices, effective participation in the world of work and support to work ability and productivity. In this scenario, occupational medicine may play a central role not only protecting the health and safety of affected works, but also promoting their overall well-being, in a Total Worker Health perspective. By recognizing work as a determinant of health, occupational medicine could also provide valuable support in defining workplace accommodations, such as the reduction of hazardous exposures or physical workload; the adoption of flexible working hours; the implementation of social support, that may be helpful in improving the well-being of asthmatic patients at work, the level of self-esteem and the perception of their work ability and productivity.

## Data Availability

The datasets used and analysed during the current study are available from the corresponding author on reasonable request.

## Abbreviations

ACT: Asthma Control Test
BEC: Blood eosinophil count
BMI: Body mass index
FeNO: Fractional exhaled nitric oxide
FEV_1_: Forced expiratory volume in the first second
FVC: Forced vital capacity
HRQoL: Health-related quality of life
M: Mean
MCS-12: Mental component summary
PCS-12: Physical component summary score
SD: Standard deviation
SF-12: Short Form Health Survey
WAI: Work Ability Index
WPAI: Work Productivity and Activity Impairment
